# *Pteridium aquilinum* (L.) Kuhn—A Review of Its Toxicology, Pharmacology, and Phytochemistry

**DOI:** 10.3390/plants15030469

**Published:** 2026-02-03

**Authors:** Hisashi Kato-Noguchi, Midori Kato

**Affiliations:** Department of Applied Biological Science, Faculty of Agriculture, Kagawa University, Miki 761-0795, Kagawa, Japan

**Keywords:** Alzheimer’s disease, carcinogen, cyanogenesis, diabetes, neuroprotective, osteoarthritis, pterosin, ptaquiloside, thiaminase

## Abstract

*Pteridium aquilinum* (L.) Kuhn, known as bracken fern, is considered a poisonous plant due to its toxic substances. This species contains toxic substances and enzymes: thiaminase and an anti-thiamine substance, which cause thiamine deficiency syndrome. Prunasin induces acute cyanide poisoning. Ptaquiloside causes haematuria, retinal atrophy, immunodeficiency, and lymphoproliferative disorders. It also induces carcinogenesis in livestock, and in animals and human cell lines. Ptaquiloside has been found in the milk of cattle, goats, and sheep that grazed on *P. aquilinum* in pastures. Ptaquiloside is water-soluble and washes away from the plants into the soil with rainwater. It has been found in streams and groundwater wells. The International Agency for Research on Cancer has classified bracken fern as a Group 2B carcinogen. However, *P. aquilinum* has long been used as a folk remedy in various regions. Several studies have identified its medicinal value and bioactive compounds with potential pharmacological activity. Pterosin B and its analogues exhibit anti-osteoarthritis, anti-Alzheimer’s disease, neuroprotective, anti-cardiomyocyte hypertrophy, anti-diabetic, and smooth muscle relaxant properties. Ptaquiloside also induces apoptosis in certain human cancer cell lines and acts as an anticancer agent. Therefore, pterosins and ptaquiloside have therapeutic properties. Other compounds, including some flavonoids and polysaccharides, act as antimicrobial, antifungal, and immunomodulatory agents. Based on their structures, it is possible to develop medicines with these therapeutic properties, particularly those containing pterosins and ptaquiloside. However, more research is needed on their use in medicinal treatments.

## 1. Introduction

**Figure 1:**
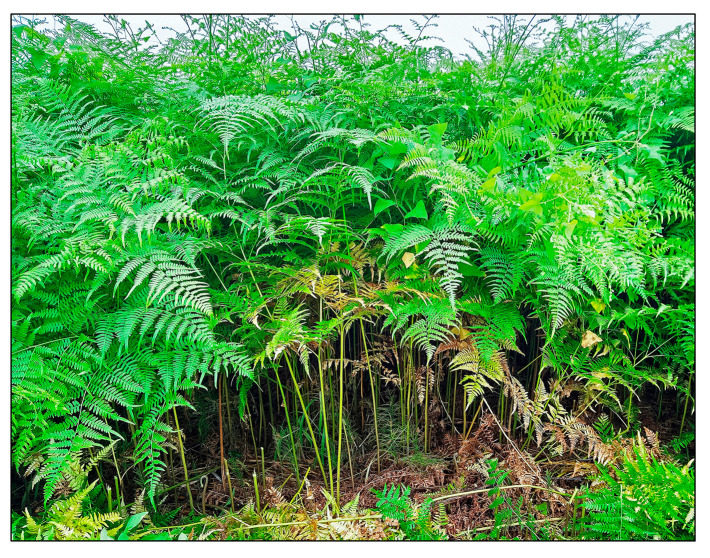
*Pteridium aquilinum* stand.

*Pteridium aquilinum* (L.) Kuhn, commonly called bracken or eagle fern, belongs to the Dennstaedtiaceae family. The genus of the *Pteridium* contains about 20 species, including *P. aquilinum* [1,2,3,4]. It is one of the oldest fern genera, with fossils dating back to the Eocene period, which occurred between 55 and 33.9 million years ago [5,6]. *Pteridium aquilinum* is a diploid species with 2*n* = 108 chromosomes [3,7]. Triploid cytotype populations of *P. aquilinum* with 2*n* = 162 chromosomes have also been recorded in various parts of Europe [8,9]. This species exhibits significant genetic diversity and phenotypic plasticity in different geographical regions and is divided into up to 12 subspecies [3,4,7]. It adapts to various environmental conditions, including temperature (low to high), rainfall, humidity, light irradiation, nutrition, soil types, and pH [7,10,11,12]. Due to its genetic diversity and adaptability, *P. aquilinum* is widely distributed in Europe, North Africa, Eastern Asia, and North and Central America [3,7]. This species thrives in open habitats, such as abandoned agricultural fields, areas burned by fire, grasslands including pastures, and coniferous and deciduous woodlands. It often forms monospecific stands [7,13,14,15] (Figure 1).

**Figure 2:**
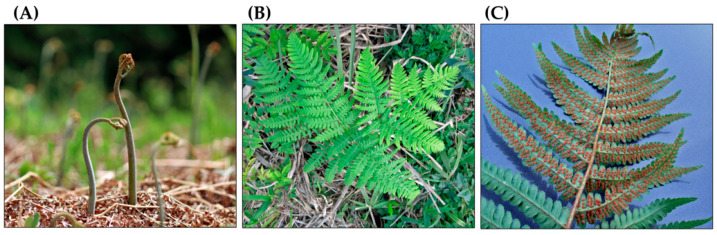
*Pteridium aquilinum*. (**A**): Fiddleheads, (**B**): Frond, (**C**): Sori (Sporangia).

As a fern species, the life cycle of *P. aquilinum* contains a large sporophyte (diploid) phase and a very small prothallium (gametophyte) phase. The prothallium is haploid and develops from spores produced by the sporophyte. The heart-shaped prothallium measures 2–5 mm in width. It produces male and female reproductive organs that generate sperm and egg cells, respectively. After featurization of the sperm and egg, the resulting diploid zygote develops into an embryo. The embryo then grows into a sporophyte [7,16,17]. The sporophyte is the dominant phase of its life cycle. It is commonly referred to as a fern and has the following structures: Fiddleheads grow directly from underground rhizomes and develop into leaves called fronds. The fronds are attached to the rhizomes by long stipes (petioles). The fronds grow between 0.3 and 3 m long. They are bipinnate and divided into pinnae (leaflets). The pinnae are further divided into pinnules. Fertile fronds produce spores in sori (sporangia), which are located on the underside of the pinnules. A single frond can produce up to 300 million spores per year [7,16,17,18]. In cold regions with harsh winters, such as Northern Europe and Eastern Asia, the fronds wither during the winter months. Their extensive, horizontal rhizomes creep underground. These rhizomes can grow up to 5 m long and 0.5 to 4 cm in diameter. They accumulate nutrients, including starch, and contribute to vegetative reproduction. Fine roots arise from the rhizomes. Sporophytes have relatively long lifespans, and their rhizomes can last up to 70 years [7,17] (Figure 2).

**Figure 3:**
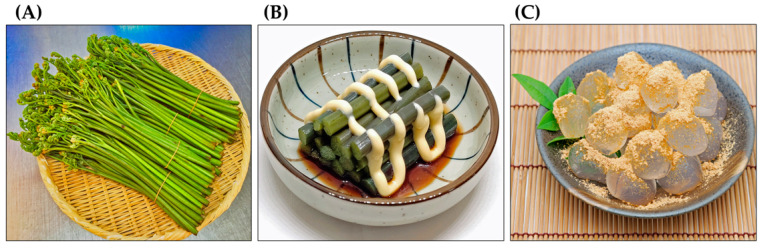
*Pteridium aquilinum* as food. (**A**): Fiddleheads at local markets, (**B**): Boiled fiddleheads with a dressing served as a vegetable, (**C**): A sweet cake made from the rhizome starch and soybean powder.

*Pteridium aquilinum* has been widely used as food and in folk medicine for a long time [19,20,21,22]. The ancient Greek pharmacologist Pedanius Dioscorides referred to *P. aquilinum* and several other ferns as having medicinal value in his book “*De materia medica*”, a pharmacopoeia of medicinal plants [23]. Decoctions made from the rhizomes of *P. aquilinum* have been used to treat diabetes and chronic disorders of the spleen and stomach. They have also been used to treat malaria and as deworming agents [24,25,26,27,28]. Decoctions made from the rhizome nodules have been used to treat rickets in children, as well as wounds, eczema, dermatitis, and coughs. They have also been used as a laxative [7,19,27]. Due to their antipyretic and diuretic properties, the fronds and rhizomes of this plant have been used to treat hepatitis [29,30]. The plant has also been used as an abortifacient for domestic animals [28,31]. The rhizomes of *P. aquilinum* contain about 45% starch. For this reason, they have been used as a food source. The rhizomes can be ground into flour and used as an ingredient in bread, cakes, and beer brewing [26,32,33,34,35,36]. The fiddleheads of *P. aquilinum* have been used as a vegetable in traditional dishes [34,36,37]. They are still available at local markets in East Asia, including China, Korea, and Japan. After proper preparation, they can be eaten as a vegetable or used as an ingredient in cooking. The starch from the rhizomes is also used to make traditional cakes (Figure 3) [29,38].

**Figure 4:**
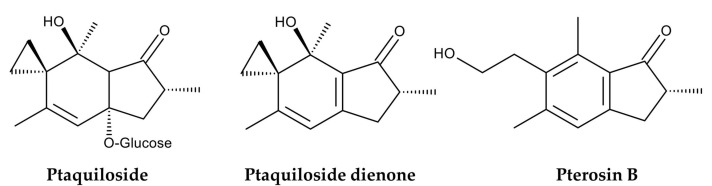
Compounds identified in *Pteridium aquilinum.*

*Pteridium aquilinum* was used as livestock feed. Dried fronds were used as cattle silage. They were mixed with hay and straw to feed horses and mules. Fresh fronds were used to feed pigs [7,26]. Since the late 19th century, the toxicity of *P. aquilinum* to livestock has been suspected intermittently [39]. Feeding on *P. aquilinum* or grazing in fields containing *P. aquilinum* often causes serious illness or death in livestock. Horses and sheep experience an acute thiamine deficiency, while cattle experience enzootic haematuria. Sheep become blind. Upper alimentary carcinoma occurs in cattle and sheep [7,32,40,41]. Extensive research has identified several toxic substances in *P. aquilinum*. One of these is ptaquiloside, a nor-sesquiterpene with highly cyanogenic properties. In vivo, it is metabolized into ptaquiloside dienone, which binds to DNA bases, such as adenine and guanine. This binding results in the cleavage and fragmentation of DNA strands. These breaks in DNA strands can induce cancer [42] (Figure 4).

The International Agency for Research on Cancer has classified bracken fern as a Group 2B carcinogen based on experimental evidence, indicating that *P. aquilinum* is possibly carcinogenic to humans [43]. Therefore, *P. aquilinum* is currently recognized as a poisonous species that threatens not only free-ranging animals but also humans who consume dairy products from livestock that feed on the ferns [41,44,45,46]. However, extracts of *P. aquilinum* have been shown to induce cell cycle arrest and apoptosis in certain human cancer cell lines [47]. Ptaquiloside also exhibited selective toxicity against human cancer cells compared to noncancer cells [48]. Furthermore, pterosin B, a degradation product of ptaquiloside (Figure 4), suppresses chondrocyte hypertrophy and protects cartilage from osteoarthritis in human cells [49] (Figure 4). Therefore, both pterosin B and ptaquiloside have therapeutic properties. This plant also contains several compounds, including some polysaccharides and flavonoids with antimicrobial, antioxidant, pesticidal, and herbicidal properties [38]. Additionally, donepezil, a substituted indanone, structural analog of pterosin B, has been approved for treating Alzheimer’s disease in the UK and USA [50]. Although consuming *P. aquilinum* is toxic*,* medicines with these therapeutic properties can be developed based on the structures of these compounds. Therefore, it is worthwhile to summarize the bioactive compounds identified in *P. aquilinum*. This review discusses the toxicology and pharmacology of *P. aquilinum* and related compounds, as well as their modes of action. A combination of online search engines was used to search the literature: Scopus, PubMed, ScienceDirect, and Google Scholar. The following terms were searched in relation to *Pteridium* and/or ptaquiloside: toxicity, pharmacology, fodder, livestock, botany, genetic diversity, habitat, ethnobotany, active compound, carcinogen, cyanogenesis, pterosin, analogue, metabolism, mode of action, occurrence, contamination, and environment. We included these research papers as thoroughly as possible. However, we excluded papers with unclear methods.

## 2. Toxicology

The toxic effects of consuming *P. aquilinum*, also known as “bracken poisoning” include thiamine (vitamin B_1_) deficiency, acute cyanide poisoning, retinal atrophy called as bright blindness, bovine enzootic haematuria, and carcinoma of the upper alimentary tract and other organs [7,32]. However, different animals exhibit different symptoms. In cattle, symptoms of bracken poisoning were observed after they consumed *P. aquilinum* for three months. These symptoms included bovine enzootic haematuria, bladder tumors, and haemorrhages from the kidneys, mucous membranes, and skin. Other symptoms were leukopenia, hyperplasia of the bile ducts, and fibrous tissue proliferation in the liver [51,52,53,54,55]. In sheep, retinal atrophy was caused by consuming *P. aquilinum* for four months. This is due to stenosis of blood vessels and degeneration of the rods, cones and outer nuclear layer of the retina [56]. Other symptoms include bone marrow suppression, immunosuppression, urinary tract neoplasia, and thiamine deficiency [57]. In horses, the most apparent symptom of bracken poisoning was thiamine deficiency caused by consuming *P. aquilinum* for three months [58,59,60]. In rats, consuming *P. aquilinum* resulted in enlarged and congested livers, renal anemia of kidney, haemorrhages, and bladder tumors [61,62]. Other symptoms include mild edema in the brain, increased intestinal secretions, and degenerative changes in hepatocytes in the liver [63]. In mice, the administration of aqueous extracts of *P. aquilinum* reduced natural killer (NK) cell activity and cellular immune responses and induced lung carcinogenesis [64,65]. Bracken poisoning has also been observed in other animals, including rabbits, pigs, and guinea pigs [66,67,68,69,70]. As mentioned in the “Introduction”, the young fronds or fiddleheads, of *P. aquilinum* are used as a vegetable in traditional East Asian cuisine. It is believed that adequate treatments, such as soaking the fiddleheads in boiling water containing wood ash or sodium bicarbonate, remove their poisonous properties [71,72,73]. The following components have been identified as the cause of “bracken poisoning”.

### 2.1. Anti-Thiamine Factors

Thiamine (vitamin B_1_) is an essential nutrient that maintains proper cellular function in humans and animals. It acts as a coenzyme in the metabolism of proteins, fats, and carbohydrates and plays a role in the central and peripheral nervous systems [74]. Thiamine deficiency leads to Wernicke-Korsakoff syndrome, and cardiovascular and neurological complications. These complications include heart failure, neuropathy, ataxia, paralysis, and delirium [75]. Descriptions of thiamine deficiency in horses were reported more than 70 years ago. Affected horses exhibited weight loss, ataxia, convulsions, and bradycardia after consuming *P. aquilinium.* Death typically occurs within two to ten days of symptom onset [59,60,76,77]. Consuming *P. aquilinum* has also been reported to cause thiamine deficiency in other animals including pigs, rats, and sheep [46,58,78].

**Figure 5:**
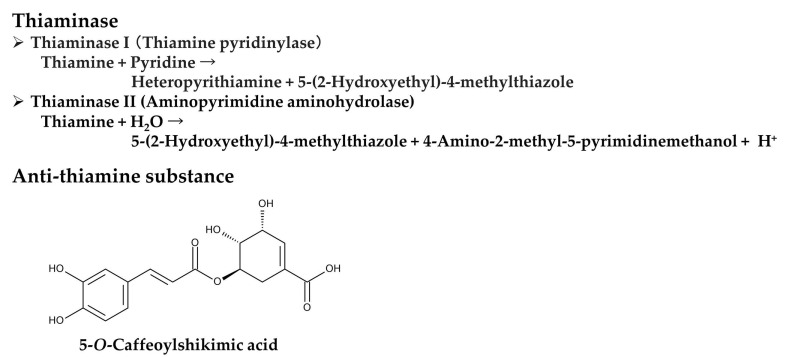
Anti-thiamine factors of *Pteridium aquilinum.*

*Pteridium aquilinium* contains two types of thiaminase: thiaminase I and thiaminase II. Thiaminase I is more active than thiaminase II in *P. aquilinium* [79]. Thiaminase I (thiamine pyridinylase, EC 2.5.1.2) catalyzes the following reaction: thiamine + pyridine → heteropyrithiamine + 5-(2-hydroxyethyl)-4-methylthiazole [80]. Thiaminase II (aminopyrimidine aminohydrolase, EC 3.5.99.2) catalyzes the following reaction: thiamine + H_2_O → 5-(2-hydroxyethyl)-4-methylthiazole + 4-amino-2-methyl-5-pyrimidinemethanol + H^+^ [81]. Therefore, consuming *P. aquilinum* results in reduced thiamine levels due to the degradation of thiamine by thiaminases I and II. This can lead to thiamine deficiency syndromes. Additionally, the extracts of *P. aquilinum* exhibited anti-thiamine activity in vitro by reducing thiamine concentrations. The active constituent was identified as 5-*O*-caffeoylshikimic acid [82]. This compound may be responsible for the anti-thiamine activity of the *P. aquilinum* extracts. However, its role in causing thiamine deficiency remains unclear (Figure 5).

### 2.2. Cyanogenic Factor

*Pteridium aquilinium* contains prunasin, a cyanogenic glycoside or a mandelonitrile glycoside [83,84,85]. Cyanogenic glycosides are toxic, and consuming these compounds has been linked to the development of various diseases due to their production of hydrogen cyanide (HCN). Hydrogen cyanide is produced through enzymatic degradation caused by plant β-glycosidases following wounding of plant tissues or by bacterial activity in the animal gut.

**Figure 6:**
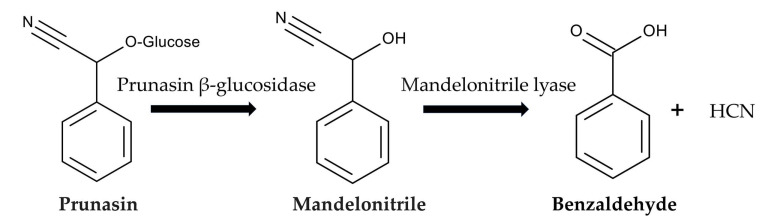
Prunasin cyanogenesis.

After ingesting *P. aquilinium,* prunasin is actively absorbed by sodium-dependent monosaccharide transporters embedded in their jejunal epithelial tissue of animals [86,87]. Then, prunasin β-glucosidase (EC 3.2.1.118) breaks it down into D-glucose and mandelonitrile. Mandelonitrile is further catalyzed by mandelonitrile lyase (EC 4.1.2.10), which produces benzaldehyde and hydrogen cyanide [88,89]. The generated hydrogen cyanide causes acute cyanide poisoning. Symptoms include low blood pressure, rapid pulse, rapid breathing, convulsions, twitching, dizziness, stupor, headaches, mental confusion, vomiting, and diarrhea [86,87] (Figure 6).

The concentration of prunasin in *P. aquilinium* was found to range from 10.4 to 61.3 mg (0.04 to 0.21 mmol) per g of fresh plant tissue [85]. Based on the metabolic pathway [88,89], one prunasin molecule (molar mass, 295.291 g) can release one hydrogen cyanide molecule (molar mass, 27.0253 g). Therefore, the maximum theoretical production rate of hydrogen cyanide is 5.7 mg (0.21 mmol) per g of fresh plant tissue.

The concentration of prunasin in the other blacken species *P. arachnoideum* was 1.8–107.7 mg (0.006–0.36 mmol) per g of dry tissue. The maximum liberation rate of hydrogen cyanide was measured at 47.9 μg per min per g of dry tissue [90]. This equates to 2.9 mg (0.11 mmol) of hydrogen cyanide released per g of dry tissue in one hour. Based on the metabolic pathway [88,89] and prunasin concentration, the maximum theoretical production rate of hydrogen cyanide is calculated to be 9.7 mg (0.36 mmol) per g of dry plant tissue. Therefore, 29.8% of the maximum rate (calculated as 2.9 mg out of 9.7 mg) was released within one hour. Applying this release rate to *P. aquilinium,* which has a maximum theoretical rate of 5.7 mg (0.21 mmol) hydrogen cyanide per g of fresh plant tissue, as described in the previous paragraph*,* results in the release of 1.7 mg of hydrogen cyanide per g of fresh tissue in one hour. The European Food Safety Authority (EFSA) established an acute reference dose (ARfD) of 20 μg of cyanide per kg of body weight [91]. This level causes acute health effects regardless of the food source [91,92]. Therefore, consuming *P. aquilinium* potentially releases enough hydrogen cyanide to cause cyanide poisoning.

Additionally, prunasin cyanogenesis increased the mortality rate of the sawfly larvae *Strongylogaster impressata* and *Strongylogaster multicincta* when they fed on *P. aquilinium* [83]. Prunasin cyanogenesis also reduced the activity of adults of the desert locust *Schistocerca gregaria* [84]. Therefore, prunasin may also act as a defense mechanism against herbivorous insects.

### 2.3. Carcinogenic Factor

#### 2.3.1. Identification of Ptaquiloside as a Carcinogenic Factor

Studies have shown that cattle that consume *P. aquilinum* develop bladder carcinoma [68,93]. Oral administration of *P. aquilinum* to rats has been shown to induce tumors in their urinary bladders and intestines [94,95,96]. Applying an extract of urine from cattle fed *P. aquilinum* to the skin of mice resulted in the growth of papilloma-type excrescences [97]. These findings indicate that *P. aquilinum* contains certain carcinogenic substances. For over 20 years, phytochemical studies have been carried out to identify the carcinogens in several blacken species.

Boiling water extracts of *P. aquilinum* were found to induce tumors in the urinary bladder and ileum of rats [72]. Ptaquiloside, an illudane-type nor-sesquiterpene glucoside, was then isolated from these boiling water extracts and identified as a carcinogenic compound [98,99] (Figure 4). Oral administration of ptaquiloside to rats has been shown to cause tumors in their urinary bladders, mammary glands, and intestines [61,100]. Intravenous and catheter administration of ptaquiloside to sheep has been shown to induce retinal atrophy [56]. Intraperitoneal administration of ptaquiloside to mice induced B-cell lymphoproliferative disorders and early-stage urothelial lesions [101]. Oral administration of ptaquiloside reduced natural killer (NK) cell activity and cellular immune responses in mice [102,103]. Additionally, a histological investigation revealed that the chronic symptom of bovine enzootic haematuria is accompanied by the development of multiple bladder tumors [54,104]. Therefore, ptaquiloside can cause retinal atrophy, lymphoproliferative disorders, immunosuppressive effects, bovine enzootic haematuria, and cancer.

Ptaquiloside has been found in several other bracken species, including *Pteridium esculentum* and *Pteridium revolutum*. These species are native to the tropical and subtropical regions of Asia and the South Pacific, including New Zealand and Australia [105,106,107]. Ptaquiloside has also been found in *Pteridium arachnoideum* and *Pteridium caudatum*, which are native to Mexico and tropical and subtropical America [108,109,110,111]. The concentration of ptaquiloside in these bracken species was similar to that in *P. aquilinum* and depended on harvest time and location. Based on these findings, it is possible that the remaining bracken species contain ptaquiloside as well. Ptaquiloside has also been found in species from other fern families, including several *Pteris* and *Onychium* species belonging to the Pteridaceae family [112,113,114]. Some ancient ferns may have acquired the ptaquiloside metabolic pathway before their families evolved. However, the evolutionary process of fern species that contain ptaquiloside remains unclear.

#### 2.3.2. Occurrence of Ptaquiloside

The concentration of ptaquiloside in *P. aquilinium* plants varied from that reported in the literature. The concentration of ptaquiloside in the fronds ranged from 0.03 to 13.3 mg per g of dry plant tissue [115,116,117,118,119]. The ptaquiloside concentration in the rhizomes was reported to be between 0.007 and 0.657 mg per g of dry plant tissue [108,115]. The ptaquiloside concentration in the spores was determined to be 0.003 mg per g of dry plant tissue [120]. Significant regional variation in ptaquiloside concentration was observed in *P. aquilinium* populations in the UK [121,122]. *Pteridium aquilinium* collected in Denmark has a higher concentration of ptaquiloside than that collected in Sweden and Finland [122]. These results imply that climate and/or geographic factors may influence the production of ptaquiloside in *P. aquilinium*. The stage of development of the fronds also affects the concentration. Young fronds contain more ptaquiloside than mature ones [115,119,123]. The production of ptaquiloside is affected by the availability of nutrients, particularly phosphate, in the soil. A high concentration of phosphate in the soil increases the production [117]. Therefore, reported differences in ptaquiloside concentration may be due to variations in harvesting time, location, and nutrient conditions. The method used to determine the concentration also affects the results [115,116,117,118,119].

Jersey-Holstein half-breed cows were fed 6 kg of fresh *P. aquilinum* daily. The ptaquiloside content of this feed ranged from 2400 to 10,000 mg. Ptaquiloside was detected in their milk 38 h after feeding. A milk sample from daily milking of 20 L contained 8.6% of the total ptaquiloside intake [124,125]. However, this estimate is quite high compared to others. The ptaquiloside concentration in milk from a dairy farm was estimated to be around 1.5, 1.8, and 1.9 μg per L for cattle, goats and sheep, respectively [126]. Ptaquiloside was also detected in the milk of goats and sheep that frequently grazed on *P. aquilinum* in several pastures in southern Italy. Concentrations of ptaquiloside ranged from the detection limit to 3.14 μg per L for goats and from the detection limit to 1.63 μg per L for sheep [127]. Although the concentration of ptaquiloside in milk varies by source, these animals excrete a certain amount of ptaquiloside into their milk by consuming *P. aquilinum*. Since ptaquiloside is carcinogenic, it is important to determine its potential toxicological effects in dairy products.

Due to its water solubility, ptaquiloside in *P. aquilinum* fronds is easily washed away by rain and leaches into the soil and groundwater. In Danish forests dominated by *P. aquilinum*, the concentration of ptaquiloside in the soil of the forest floor was 1600 mg per m^2^. A single rainfall event washed 13.2 mg per m^2^ (2.5 mg per L) of ptaquiloside into the soil. The concentration of ptaquiloside in soil water was measured between 4.4 and 4.8 μg per L [123,128]. In a small stream draining a *P. aquilinum*-infested catchment area in the UK, the ptaquiloside concentration was up to 0.06 μg per L. During storm events, this concentration increased to 2.2 μg per L [129]. The concentration of ptaquiloside in groundwater wells (ranging from 8 to 40 m deep) in regions dominated by *P. aquilinum* in Denmark, Sweden, and Spain was determined. Six of the seven wells containing ptaquiloside were used for drinking water, with concentrations ranging from 0.27 to 0.75 μg per L [130]. Ptaquiloside was detected at all the water abstraction sites examined in Northern Ireland, where *P. aquilinum* is prevalent. The highest concentration found was 0.67 μg per L in drinking water [131]. Further research is needed to determine the potential toxicological effects of ptaquiloside in drinking water in areas where *P. aquilinum* grows abundantly.

#### 2.3.3. Metabolism of Ptaquiloside

Ptaquilosin, the aglycone of ptaquiloside, was synthesized artificially from dimethyl (1*R*,2*R*)-cyclopentane-1,2-dicarboxylate in 20 steps with a total yielding of 2.9% [132]. However, the biosynthesis of ptaquiloside is not fully understood. It may be synthesized from farnesyl pyrophosphate, which is a precursor of sesquiterpenes. Farnesyl pyrophosphate undergoes cyclization, oxidative cleavage, and glycosylation [133]. The spirocyclopropane ring in ptaquiloside makes the compound susceptible to reactions such as ring opening, rearrangement, and addition [134].

**Figure 7:**
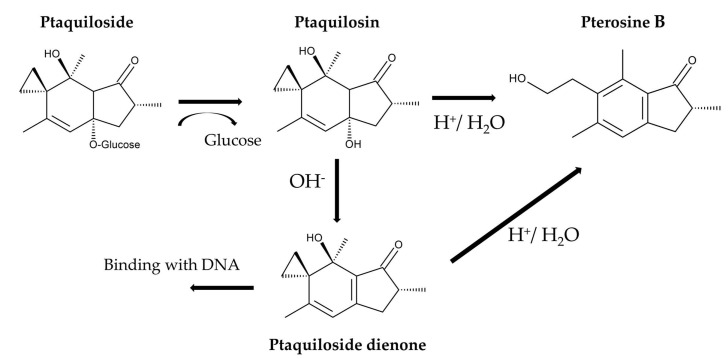
Ptaquiloside metabolism.

Ptaquiloside degradation in water is pH-dependent [135]. Under neutral conditions, ptaquiloside is relatively stable. Under alkaline conditions, ptaquiloside is attacked by OH^−^ and breaks down into ptaquilosin and glucose. Ptaquilosin then converts into a dienone intermediate, which is highly unstable. This intermediate either covalently binds to DNA molecules or forms pterosin B with water [39,133,136]. Under acidic conditions, protonation and tautomerization occur in the ptaquiloside molecule. The cyclopropane ring of ptaquiloside undergoes nucleophilic attack by H^+^, which leads to aromatization with glucose elimination, and ring opening of the spirocyclopropane. This results in the formation of pterosin B. This process does not produce a dienone intermediate [41,137,138]. Ptaquiloside degradation was slow at pH 5.5 and increased below pH 4.3. The half-life of ptaquiloside was 170 min at pH 2.0 [139,140]. Additionally, ptaquiloside was metabolized into pterosin B in fresh cattle rumen solution [137] (Figure 7).

The concentrations of pterosin in the fronds were determined by LC-MS to confirm the metabolism of ptaquiloside in *P. aquilinum.* The concentrations of ptaquiloside and pterosin B in *P. aquilinum* fronds were between 0 and 6.3 and between 0 and 0.45 mg per g of dry plant tissue, respectively [119]. Another study using LC-MS quantification found ptaquiloside concentrations ranging from 0 to 13.7 mg per g of dry plant tissue and pterosin B concentrations ranging from 0.004 to 0.73 mg per g of dry plant tissue. The recovery rate for both compounds during the determination process was over 86% [118]. These results suggest the metabolism of ptaquiloside into pterosin B in *P. aquilinum* fronds. However, the concentrations of these compounds and their ratio vary depending on the sampling location [118,119].

When 0.21 mg of ptaquiloside per kg of body weight were administrated intravenously to cattle, the concentration of ptaquiloside in their plasma decreased rapidly, and fell below the detection limit after 12 h. Conversely, ptaquiloside was detected in their urine shortly after administration. Most of the ptaquiloside excreted into the urine occurred within the first four hours after administration [137]. These results suggest that ptaquiloside is quickly excreted into the urine after entering the bloodstream. When cattle were administrated *P. aquilinum* powder orally at a dose of 1.6 mg of ptaquiloside per kg of body weight, pterosin B was detected in their plasma 10 min afterward and remained detectable up to 12 h later. The maximum concentration of pterosin B in the plasma was 39.7 μg per L at one hour after administration. Shortly after administration, pterosin B was also detected in the urine of cattle. Up to 7% of the pterosin B equivalent of ptaquiloside was excreted into the urine within 24 h after administration. Ptaquiloside was not detected in the plasma and urine at any time [137]. These findings from the oral administration of *P. aquilinum* powder suggest that ptaquiloside is rapidly metabolized into pterosin B and not absorbed into the bloodstream. The produced pterosin B may then be quickly absorbed into the bloodstream and excreted in the urine. However, only 7% of the equivalent amount of ptaquiloside was excreted in the urine as pterosin B. The rest is likely metabolized into other compounds, such as ptaquiloside diene, and absorbed by certain organs in cattle. The complete metabolism of ptaquiloside in animals following the oral administration of *Peridium aquilinum* remains unclear.

**Figure 8:**
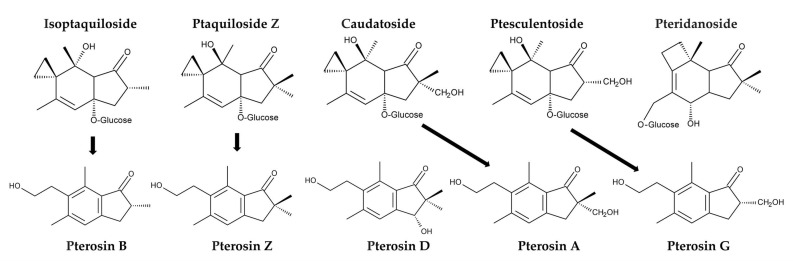
Ptaquiloside and pterosin analogues identified in *Pteridium aquilinum.*

Additionally, *P. aquilinum* was found to contain several other illudane-type sesquiterpene glucosides, including isoptaquiloside, ptaquiloside Z (methylptaquiloside), caudatoside, and ptesculentoside. It also contains the protoilludane-type sesquiterpene glucoside pteridanoside [109,118,138,141,142,143]. Their corresponding pterosins, pterosin B, pterosin Z, pterosin A, and pterosin G, were identified [118,138,144]. Pterosin D, a C_3_-hydroxylated form of pterosin Z, was also identified [145]. However, information on the metabolism and biological activities of these analogues is limited (Figure 8).

#### 2.3.4. Mode of Action of Ptaquiloside

**Figure 9:**
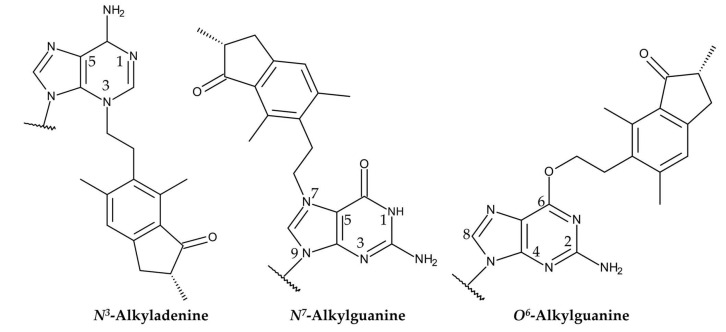
Structures of alkylated adenine and guanine formed in a reaction with ptaquiloside dienone.

Ptaquiloside contains a spirocyclopropane moiety adjacent to a hydroxy group within its cyclohexene unit. The spirocyclopropane moiety is electrophilic and highly reactive [134]. The carcinogenicity of ptaquiloside is attributed to its dienone metabolites with a spirocyclopropane ring [45,133,136,146]. Ptaquiloside dinene induces alkylation of heterocyclic ribonucleotide bases, particularly adenine and guanine. This results in the formation of *N*^3^-alkyladenine and *N*^7^-alkylguanine adducts, respectively [147,148]. The formation of *O*^6^-alkylguanine adducts has also recently been reported. This alkylation is resistant to the DNA repair enzyme *O*^6^-alkylguanine-DNA methyltransferase (MGMT) [146] (Figure 9).

The alkylation is followed by adducted DNA hydrolysis, spontaneous depurination and DNA chain cleavage at the resulting abasic sites via a β-elimination response [136,146]. DNA chain cleavage occurs selectively at the adenine site during the initial response period. Subsequently, cleavage occurs at the guanine site of the DNA strands at a later time [148,149]. The most favorable abasic sites, indicating **A**, on DNA strands were found to be the base sequences 5′-AA**A**TA and 5′-AT**A**TA [148,149,150]. These abasic sites of the DNA strands can be mutagenic, resulting in the induction of carcinogenesis [45,133,136,146,151].

Ptaquiloside can also target and reduce the functionality of cytotoxic CD8^+^ T cells and natural killer (NK) cells [102,152]. These cells are involved in immune function and eliminate infected viral, neoplastic and damaged cells by inducing apoptosis in target cells [153,154]. A reduction in cytotoxic immune function increases the risk of oncogenic viral infection. Mutagenic agents can also accelerate carcinogenesis [155,156]. Ptaquiloside promotes the development of papillomavirus-related cancers of the upper digestive tract, oropharynx, and oral cavity in vivo [157,158]. Therefore, ptaquiloside may work with papillomavirus to induce carcinogenesis. These findings suggest that ptaquiloside induces carcinogenesis by accelerating virus oncogenesis and alkylating DNA. This occurs by reducing the functionality of immune cells, such as natural killer cells and cytotoxic CD8^+^ T cells. However, the mechanism of ptaquiloside on the reduction in the functionality of these cells remains unclear.

## 3. Pharmacology

As mentioned in the “Introduction”, *P. aquilinum* has been used in traditional folk medicines [19,20,21,22]. Several studies have identified its medicinal value and bioactive compounds with potential pharmacological activity.

### 3.1. Antimicrobial and Antiviral Properties

Oral administration of 400–800 mg per kg of body weight of methanol extracts of *P. aquilinum* fronds to malaria-infected mice demonstrated an antimalarial effect, reducing *Plasmodium berghei* parasites by 8.7–79.5% [159]. In an in vitro assay, aqueous ethanol extracts of *P. aquilinum* rhizomes exhibited SARS-CoV-2 viral growth inhibitory activity with an EC_50_ of 7.43 μg per mL [160].

**Figure 10:**
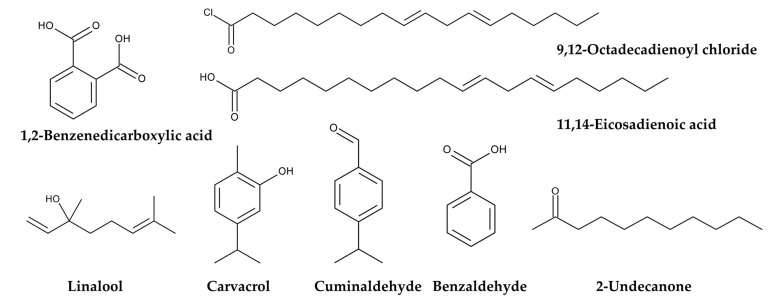
Antimicrobial compounds identified in *Pteridium aquilinum.*

Ethanol, petroleum ether, and/or *n*-hexane extracts of *P. aquilinum* fronds exhibited antimicrobial activity against the following bacteria: *Staphylococcus aureus*, *Bacillus subtilis*, *Escherichia coli*, *Proteus vulgaris*, and *Enterobacter aerogenes* [161,162]. Methanol extracts of *P. aquilinum* fronds exhibited antifungal activity against *Candida albicans* and *Aspergillus niger,* and antibacterial activity against *S. aureus*, *Salmonella typhimurium*, *E. coli*, and *Pseudomonas aeruginosa*. The active components identified in the extracts were 1,2-benzenedicarboxylic acid, 9,12-octadecadienoyl chloride, and 11,14-eicosadienoic acid [163]. Essential oil obtained from *P. aquilinum* fronds exhibited antibacterial activity against *Erwinia amylovora*, *Pectobacterium carotovorum*, and *Pseudomonas savastanoi*. The major components and their respective masses were linalool (10.29%), carvacrol (8.15%), benzaldehyde (5.95%), 2-undecanone (5.32%), and cuminaldehyde (4.57%) [164] (Figure 10). Therefore, the extracts have antimicrobial and antiviral activities, as well as anti-malaria and anti-SARS-CoV-2 activities.

### 3.2. Antioxidant, Anti-Inflammatory and Immunomodulatory Properties

**Figure 11:**
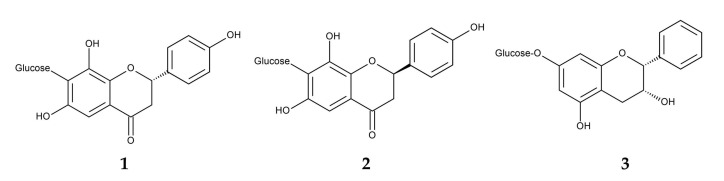
Anti-inflammatory compounds identified in *Pteridium aquilinum.* **1**: (*S*)-4′,6,8-Trihydroxyflavanone-7-*C*-glucoside, **2**: (*R*)-4′,6,8-Trihydroxyflavanone-7-*C*-glucoside, **3**: Distenin-7-*O*-*β*-d-glucoside.

Flavonoid-rich extracts from the fronds of *P. aquilinum* have demonstrated antioxidant activity [165]. Several flavonoids have been isolated from fronds of *P. aquilinum*, including the newly identified (*S*)-4′,6,8-trihydroxyflavanone-7-*C*-glucoside, (*R*)-4′,6,8-trihydroxyflavanone-7-*C*-glucoside, and distenin-7-*O*-*β*-d-glucoside. These flavonoids exhibited anti-inflammatory activity [166] (Figure 11).

The fronds of *P. aquilinum* contain polysaccharides with antioxidant properties. One such polysaccharide consists of the following sugars and their respective masses: glucose (58.1%), galactose (18.7%), rhamnose (10.2%), mannose (6.8%), and arabinose (6.1%). This polysaccharide has an average molecular weight of 458,000 Da. At a concentration of 800 μg per mL, this polysaccharide exhibited 83.1% DPPH radical scavenging activity in vitro [167]. Another polysaccharide consists of the following sugars at the indicated ratios: mannose (4.81), galactose (4.57), xylose (3.33), fucose (3.26), arabinose (1.58), and rhamnose (1.00). Its average molecular weight is 214,000 Da. This polysaccharide exhibited 98.8% DPPH radical scavenging activity at a concentration of 2000 μg per mL. It also demonstrated significant immunomodulatory activity. It induced the proliferation of mouse monocyte macrophages (RAW264.7) at concentrations ranging from 12.5 to 200 μg per mL, which indicates its immunomodulatory activity [168]. Therefore, these polysaccharides possess antioxidant and immunomodulatory properties. However, the active principles in the flavonoid-rich extracts responsible for the antioxidant activity remain unclear.

### 3.3. Antidepressant Property

Oral administration of flavonoid-rich *P. aquilinum* extracts produced antidepressant-like effects in mice. The extracts significantly reduced stress-induced immobility time and increased activity time. The extracts increased serotonin and dopamine levels in the hippocampus of the mice. The extracts also exhibited antioxidant and anti-inflammatory properties, protecting against neuroinflammation and oxidative damage caused by the underlying factors of chronic stress and depression [169]. Therefore, the extracts have antidepressant properties. However, the active principles among the flavonoids remain unclear.

### 3.4. Antidiarrheal Property

Oral administration of 245–735 mg per kg of body weight of methanol extracts from *P. aquilinum* fronds reduced castor oil-induced diarrhea in mice by 74.2–80.5% [170]. The active principles in the methanol extracts that are responsible for the antidiarrheal activity are unclear.

### 3.5. Anticancer Effects and Ptaquiloside

Dichloromethane extracts from *P. aquilinum* fronds induced apoptosis in human cancer cell lines, including transitional cell carcinoma (TCC), embryonal carcinoma (NTERA2), and breast adenocarcinoma (MCF-7) cells. The extracts also induced cell cycle arrest between the G2 and M phases in these cells [47]. Ptaquiloside exhibited anticancer activity against several human cancer cell lines, including colon tumor (HCT116), pancreatic carcinoma (MIA PaCa-2), and neuroblastoma (SK-N-AS) cells. The IC_50_ values for cytotoxicity against these cancer cells range from 22 to 70 μM. However, ptaquiloside exhibited lower cytotoxic activity against non-cancer human retinal epithelial (ARPE-19) cells, with IC_50_ values exceeding 100 μM [48]. These results suggest that *P. aquilinum* extracts and ptaquiloside may have anticancer properties. However, as described in Section 2.3, ptaquiloside is carcinogenic. Further investigation is needed regarding their use in medicinal treatments. This includes determining the appropriate ptaquiloside concentration, identifying the cancer cell lines to which it will be applied, and assessing its safety.

### 3.6. Pterosin Pharmacology

The ptaquiloside metabolite pterosin B has been reported to exhibit no significant carcinogenic or cytotoxic activity [171,172,173]. Pterosin B and its analogues have demonstrated several pharmacological properties that could be useful in treating osteoarthritis, Alzheimer’s disease, cardiomyocyte hypertrophy, and diabetes. These compounds have also exhibited neuroprotective and smooth muscle relaxant properties.

#### 3.6.1. Anti-Osteoarthritis Property

Osteoarthritis is a common and debilitating joint disorder, affecting over 350 million people worldwide. It is characterized by inflammation, thinning of the articular cartilage, formation of osteophytes, and changes to the subchondral bone [174,175]. Salt-inducible kinase 3 (Sik3) promotes the development of osteoarthritic cartilage in humans [176]. Therefore, Sik3 is a potential target for osteoarthritis treatment. Pterosin B suppressed the Sik3 signaling pathway and inhibited the hypertrophy in human chondrocytes, thereby protecting cartilage from osteoarthritis in vitro [49]. Pterosin B also suppressed the expression of hypertrophy markers, including matrix metalloproteinase 13 (MMP13) and type X collagen, in human osteoarthritic cartilage cells [177]. These findings suggest that pterosin B protects osteoarthritic cartilage by suppressing Sik3 signaling, expression of MMP13, and accumulation of type X collagen. Therefore, pterosin B could be a therapeutic candidate for treating osteoarthritis.

#### 3.6.2. Anti-Alzheimer’s Diseases and Neuroprotective Properties

Alzheimer’s disease is an irreversible, age-related brain disorder, affecting over 55 million people worldwide. It is characterized by memory impairment, behavioral disturbances, and cognitive dysfunction. The accumulation and oligomerization of the amyloid β-peptide in the brain play a significant role in the pathogenesis of Alzheimer’s disease [178,179]. The overproduction of the amyloid β-peptide is induced by the β-site amyloid precursor protein-cleaving enzyme 1 (BACE1), which is followed by oligomerization. This process leads to neurodegeneration [180]. Increased BACE1 activity increases the risk of traumatic brain injury, cardiovascular events, and stroke [181]. Therefore, BACE1 is a potential therapeutic target, and BACE1 inhibitors are a possible treatment for Alzheimer’s disease [182]. Decreased levels of acetylcholine in the brain also play a significant role in the pathogenesis of Alzheimer’s disease [179,183]. Acetylcholine is a neurotransmitter involved in cognitive processes. It is broken down by acetylcholinesterase (AChE) and butyrylcholinesterase (BChE). This breakdown results in a loss of cognitive function [179,183]. These enzymes are also therapeutic targets for treating the cognitive deficits associated with the pathogenesis of Alzheimer’s disease [184].

**Figure 12:**
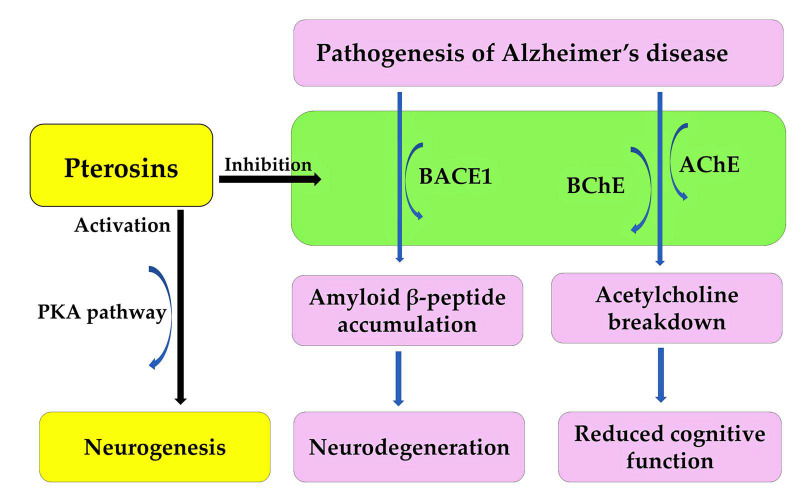
Pterosin B acts as an anti-Alzheimer’s disease agent.

Pterosin B demonstrated high blood–brain barrier permeability and significantly reduced the secretion of amyloid β-peptide from neuroblastoma cells. It inhibited the activities of BACE1, AChE, and BChE, with respective IC_50_ values of 29.6, 16.2, and 48.1 μM. Pterosin B is a noncompetitive inhibitor of human BACE1 and BChE, and a mixed-type inhibitor of human AChE. It binds to the active sites of these enzymes [185]. Pterosin B has been shown to have significant neuroprotective activity against glutamate excitotoxicity [173], and to improve cognitive deficits in Alzheimer’s disease by reducing neuroinflammation [186]. Therefore, pterosin B can target multiple therapeutic agents, including BACE1, AChE, and BChE. It can also protect against neuroinflammation during the pathogenesis of Alzheimer’s disease. Pterosin B analogues, such as pterosin A and Z, inhibited BACE1, AChE and/or BChE activity. However, pterosin B exhibited the greatest inhibitory activity [185]. Additionally, pterosin D significantly improved the cognition and memory of 5xFAD Alzheimer’s model mice by activating the protein kinase A (PKA) pathway [187]. PKA is involved in memory formation and neurogenesis [188]. Together, these findings suggest that pterosin B and its analogues could be used to treat the pathogenesis of Alzheimer’s disease (Figure 12).

#### 3.6.3. Anti-Cardiomyocyte Hypertrophy Property

Pterosin B reduced hypertrophy related genes expression, protein synthesis and cell size in a rat embryonic heart-derived cell line (H9c2 cells). Under angiotensin II stimulation, pterosin B reduced the activation of the angiotensin II type 1 receptor by inhibiting the phosphorylation of the PKC-ERK-NF-κB pathway. This pathway consists of protein kinase C (PKC), extracellular signal-regulated kinase (ERK), and **n**uclear factor kappa-light-chain-enhancer of activated B cells (NF-κB) [189]. Angiotensin II increases blood pressure and induces cardiomyocyte hypertrophy and arteriosclerosis [190]. These results suggest that pterosin B can inactivate the angiotensin receptor and protect against these conditions. Therefore, pterosin B is a potential therapeutic candidate for treating cardiomyocyte hypertrophy and arteriosclerosis.

#### 3.6.4. Anti-Diabetic Property

Oral administration of pterosin A to diabetic mice improved their hyperglycemia and glucose intolerance, while decreasing their serum insulin levels and insulin resistance. Pterosin A increased AMP-activated protein kinase (AMPK) and GLUT4 translocation from the cytosol to the membrane in skeletal muscles, while decreasing phosphoenolpyruvate carboxyl kinase (PEPCK) expression in the livers of these mice. In human hepatic cells, pterosin A increased intracellular glycogen levels and decreased PEPCK expression and glycogen synthase. In cultured human muscle cells, pterosin A increased glucose uptake and AMPK phosphorylation [191]. Therefore, pterosin A may inhibit glycogenesis in the liver while promoting glucose consumption in muscles. Pterosin A protects rat pancreatic insulin-secreting (RINm5F) cells against oxidative stress and palmitate-induced damage via the AMPK signaling pathway [192]. These results suggest that pterosin A may be an effective therapeutic option for treating diabetes.

#### 3.6.5. Smooth Muscle Relaxant Property

Pterosin Z exhibited smooth muscle relaxant activity by inhibiting calcium influx in potassium-depolarized guinea pig ileum tissues in a concentration-dependent manner, with an EC_50_ of 1.3 μM. This treatment resulted in muscle relaxation in the tissues [193]. Smooth muscle relaxants relieve tightness and spasms in involuntary muscles in the digestive, respiratory, vascular, and urinary systems. These medications are used to treat conditions such as irritable bowel syndrome, high blood pressure, asthma, and cramps [194]. Therefore, pterosin Z has a smooth muscle relaxant property.

## 4. Conclusions

*Pteridium aquilinum* contains several toxic enzymes and substances. Thiaminase I and II, and an anti-thiamine substance cause thiamine deficiency syndrome. Prunasin induces acute cyanide poisoning. Ptaquiloside causes haematuria, retinal atrophy, immunodeficiency, and lymphoproliferative disorders, and cancer in livestock, and/or animals and human cell lines. Ptaquiloside was found not only found in *P. aquilinum* and other *Pteridium* species*,* but also in the milk of livestock that grazed *P. aquilinum* in pastures and in the drinking water of areas where *P. aquilinum* grows abundantly.

*Pteridium aquilinum* also contains several pharmacologically active substances. Pterosin B and its analogues exhibit anti-osteoarthritis, anti-Alzheimer’s disease, neuroprotective, anti-cardiomyocyte hypertrophy, anti-diabetic, and/or smooth muscle relaxant properties. Ptaquiloside is not only carcinogenic, but also induces apoptosis and suppresses carcinogenesis in certain human cancer cell lines. Other compounds act as antimicrobial, antifungal, and immunomodulatory agents. Based on their structures, medicines with these therapeutic properties can be developed, particularly those containing pterosins and ptaquiloside. Further investigation is needed regarding their use in medicinal treatments. This includes determining the appropriate concentration, identifying the cell lines to which they will be applied, and evaluating their safety in vitro and in vivo conditions.

## Data Availability

No new data were created or analyzed in this study.
